# MGlu5 Dependent Mitochondrial Translocation of PKCδ: A Mechanism Raising Astrocytic Oxidative Metabolism in Response to Extracellular Glutamate

**DOI:** 10.1111/jnc.70163

**Published:** 2025-07-30

**Authors:** Kiavasch M. N. Farid, Rodrigo Lerchundi, Christine R. Rose, Amin Derouiche

**Affiliations:** ^1^ Institute of Anatomy II Goethe University Frankfurt Frankfurt am Main Germany; ^2^ Institute of Neurobiology Heinrich Heine University Düsseldorf Düsseldorf Germany

**Keywords:** glia‐synaptic interaction, neurometabolic coupling, pyruvate dehydrogenase, pyruvate dehydrogenase phosphatase, tripartite synapse

## Abstract

Synaptic activity imposes high demands of local energy production on astrocytes. However, the (an)aerobic pathways and fuel for generation of energy equivalents in astrocytes are still debated. Also, mechanisms to ensure rapid metabolic adaptation to bouts of neuronal activity have not been sufficiently explored. Here, we show a mechanism in astrocytes linking extracellular glutamate to upregulation of oxidative phosphorylation. We stimulated primary astrocytes with glutamate, and applied fluorescent immunocytochemistry with anti‐protein kinase Cδ (PKCδ), anti‐pyruvate dehydrogenase (PDH) and anti‐phospho‐PDH antibodies, and object oriented image analysis. Glutamate induces mitochondrial translocation of PKCδ and subsequent activation of the mitochondrial enzyme PDH—the point‐of‐no‐return in the utilization of carbohydrates. Using the specific mGlu5 antagonist 2‐Methyl‐6‐(phenylethynyl)pyridine hydrochloride (MPEP), the metabotropic glutamate receptor 5 (mGlu5) was identified as the key receptor inducing mitochondrial PKCδ translocation and PDH activation. We demonstrate by luminometric ATP assay and subtype‐specific inhibitors of PKC and mGlu5 that the distinct initial drop in intracellular ATP following glutamate application is counteracted by the mGlu5/PKCδ‐dependent mitochondrial activation. mGlu5 inhibition decreases ATP production also in astrocytes in the acute brain slice. Collectively, these findings reveal that astrocytes possess a potential for oxidative phosphorylation that can be stimulated by extracellular glutamate and the mGlu5/PKCδ/PDH axis, suggesting targets for pathologies involving excess glutamate. This also focuses the issue of activity‐induced glia‐neuronal metabolic interaction on perisynaptic energetics and the glia‐*synaptic* microenvironment. Up‐regulation of astrocytic metabolism via the mGlu5/PKCδ/PDH axis may affect only those perisynaptic astrocyte processes (PAPs) close to the active synapse(s), leaving other astrocyte domains and the whole cell unchanged.

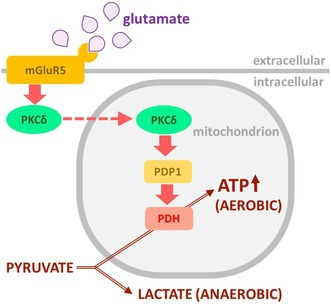

## Graphical abstract

The observations suggest a new mechanism, the mGluR5/PKCδ/PDH axis by which extracellular glutamate can raise oxidative metabolism in astrocytes, in response to rapidly increasing energy demands, such as in the liver or skeletal muscle. It appears that the plethora of astrocytic mitochondria maintain only a basal level of oxidative metabolism during low synaptic activity. The appearance of glutamate in the extracellular space, as under high activity of glutamatergic synapses, would trigger this mechanism, shifting astrocytes towards oxidative metabolism. This represents a considerable stand‐by potential for add‐on capacity that can be stimulated by extracellular glutamate.

**Abbreviations**
APV: (2R)‐amino‐5‐phosphonovaleric acid
CMOS: complementary metal oxide semiconductor
COX: cytochrome c oxidase
DMEM: Dulbecco's modified Eagles Medium
DPBS: Dulbecco's Phosphate‐Buffered Saline
FRET: Förster resonance energy transfer
GFAP: glial fibrillary acidic protein
IDR: intensity density ratio
IQR: interquartile range
MEM: minimum essential medium
mGlu5: metabotropic glutamate receptor 5
MPEP: 2‐Methyl‐6‐(phenylethynyl)pyridine hydrochloride
NBQX: 2,3‐dihydroxy‐6‐nitro‐7‐sulfamoyl‐benzo[f]quinoxaline
NHS: normal horse serum
PAP: peripheral astrocyte processes
PB: phosphate buffer
PDC: pyruvate dehydrogenase complex
PDH: pyruvate dehydrogenase
PDK: pyruvate dehydrogenase‐kinase
PDP: pyruvate dehydrogenase phosphatase
PKC: protein kinase C
PKCδ: protein kinase Cδ (delta)
p‐PDH: phospho‐Ser293 pyruvate dehydrogenase
ROI: region of interest
RRID: Research Resource Identifier (see scicrunch.org)
RT: room temperature
TTX: tetrodotoxin

## 1 Introduction

Astrocytes and their metabolism are essential for energy homeostasis of the brain (Barros et al. 2018). Their metabolism subserves energy supply to neurons (Pellerin and Magistretti 2012), and they also accomplish neurotransmitter uptake and recycling, processes that are ATP‐dependent (Dienel 2013). As a corollary, energy requirements are particularly high in relation to neuronal activity and neurotransmission; thus, rapid metabolic regulation is key for activity‐dependent function. Arising from this are the two highly studied issues on (inter‐)cellular energetics: How, in what form, and from what fuel do astrocytes generate energy equivalents; and how do astrocytes ensure rapid metabolic adaptation to neuronal activity?

There has been considerable controversy whether astrocytes mainly employ glycolytic or oxidative pathways to cover their energy requirements. Following one line of evidence, lactate is glycolytically generated from glycogen or glucose, and transferred to neurons (Pellerin and Magistretti 1994) (reviewed by (Pellerin and Magistretti 2012; Barros et al. 2023)), a setting assuming very limited oxidative energy production in astrocytes, and recycling of most transmitter glutamate as glutamine to neurons. A coherent series of other findings (reviewed by (Dienel et al. 2023; Lovatt et al. 2007)) assumes that astrocytes possess significant, underestimated oxidative metabolic capability, which leads to efficient ATP production. The transmitter glutamate, an integral component of this metabolism, is only partially recycled, the other part fueling the TCA cycle and oxidative ATP generation (McKenna 2013).

To acutely adjust their energy production to the demands of glutamatergic synaptic activity, astrocytes might employ several mechanisms triggered by extracellular glutamate. For example, glutamate may signal via glial high‐affinity glutamate uptake to increase energy production via various downstream mechanisms and pathways (Pellerin and Magistretti 1994; Langer and Rose 2009; Langer et al. 2017). Also, the increase in extracellular potassium concentration associated with neuronal activity stimulates glycolysis in astrocytes (Ruminot et al. 2011; Magistretti and Allaman 2015). Moreover, it has previously been shown that small mitochondria are positioned even in the peripheral astrocyte processes (PAPs) which are close to synapses (Derouiche et al. 2015; Owens et al. 2015; Petit and Magistretti 2016) and might thus fuel metabolism of transmitter glutamate via oxidative phosphorylation, the most efficient form of ATP generation. Petit and Magistretti (Petit and Magistretti 2016) have however doubted this assumption arguing that the activity of pyruvate dehydrogenase (PDH) in glial mitochondria is low (Halim et al. 2010).

PDH, as part of the pyruvate dehydrogenase complex, represents the “point‐of‐no‐return” in the utilization of carbohydrates, since their conversion by PDH invariably and ultimately leads to their utilization for oxidative phosphorylation and ATP generation (Denton et al. 1996). PDH plays a central role in the regulation of mammalian metabolism, and its activity is highly regulated. In astrocytes, PDH can be stimulated to 300% of its basal activity (Halim et al. 2010). In skeletal muscle and liver, that is, in tissues adapting rapidly to short term physiological requirements, PDH upregulation is mediated by hormones binding to membrane receptors. The present study investigates whether in astrocytes, a similar “on demand” adaptation of PDH activity may be mediated by extracellular glutamate.

To ensure locally regulated and quickly available ATP supply, astrocytes are able to modulate the metabolic activity of their numerous mitochondria alongside their positioning (Jackson et al. 2014). These are thought to harbor a vast oxidative capability which may fuel transmitter metabolism (especially for glutamate metabolism) (Lovatt et al. 2007; Derouiche et al. 2015; Owens et al. 2015).

Protein kinase Cδ (PKCδ) is known to modulate PDH activity in hepatocytes, so as to mediate insulin‐induced mitochondrial activity increase (Caruso et al. 2001). We investigate an analogous role of glutamate in astrocytes, where PKCδ regulates mitochondrial oxidative metabolism at the level of PDH. Our data show that extracellular glutamate can increase PDH activity and mitochondrial oxidative metabolism in astrocytes through stimulation of metabotropic glutamate receptor 5 (mGlu5) and mitochondrial PKCδ translocation. Activity‐induced up‐regulation of astrocytic metabolism via mGlu5/PKCδ/PDH may thus affect only those PAPs close to the active synapse(s), leaving other astrocyte domains and the whole cell unchanged. This focuses the issue of activity‐induced glia‐neuronal interaction on the glia‐*synaptic* micro‐environment.

## 2 Methods

### 2.1 Animal Welfare

Following the recommendations of the European Commission (Close et al. 1997), animals up to 10 days old were quickly decapitated to prepare primary astrocyte culture or organotypic brain tissue slice cultures. Experiments and sacrifice were carried out in strict accordance with the institutional guidelines (Goethe University, Frankfurt, and Heinrich Heine University Düsseldorf) as well as the European Community Council Directive (2010/63/EU). All experiments on organotypic brain tissue slice cultures were approved by the Animal Welfare Office at the Animal Care and Use Facility of the Heinrich Heine University Düsseldorf (institutional act number O50/05). All animals used in this study had access ad lib to food and water. Adult mice were of both sexes and kept with max. five cage companions in Type 2 long cages. Rat pups (of both sexes) were taken from the litter and dam kept in Type GR1800 double‐decker cages (Tecniplast, Buguggiate, Italy), without cage companions. Altogether, 40 animals were sacrificed for this study.

### 2.2 Primary Astrocyte Culture

Primary astrocyte cultures were prepared following a slightly modified version of the procedure described by (McCarthy and de Vellis 1980). Briefly, for one preparation, the cortices from two rat pups 3 days old (Sprague Dawley, of either sex; Charles River, Cologne, Germany) were dissociated by trypsinization, the cells obtained were cultured in four 75 cm^2^ flasks, at 37°C and 5% CO_2_ in air, using Dulbecco's modified Eagles Medium (DMEM; Sigma‐Aldrich, Munich, Germany, cat. no. D6046, glucose, 5.5.mM) and fetal bovine serum (10%; heat inactivated, Sigma‐Aldrich, cat. no. F7524). After 5 days in vitro, medium changes were without serum. After 7 days, most non‐astrocytic cells were shaken off using a rotary shaker (18 h, 180 rpm) and discarded. At about 13–15 days in vitro, astrocytes were replated on poly‐l‐lysine (PLL; Sigma‐Aldrich, cat. no. P6282)‐coated coverslips in 10 cm Petri dishes for immunostaining, or in PLL‐coated well plates for cell lysis and luminometric ATP measurements. Cells were replated in 24‐well plates (2.5 × 10^4^ cells/well) or 96‐well plates (0.25–1 × 10^4^ cells/well). Cell density at seeding was identical within a given plate, and luminometric ATP measurements of treated wells were related to untreated ones on the same plate, thus controlling for cell density, incubation times, and handling.

Subconfluent cells were fixed (10 min, 4% formaldehyde in 0.1M phosphate buffer, pH 7.4 [PB]) after experimental incubation, for immunostaining on day in vitro 17–21, or were lysed 2 days after replating (0.5% trichloroacetic acid [Sigma‐Aldrich, cat. no. 27242] in double distilled water, 0°C, 7 min).

### 2.3 Organotypic Brain Tissue Slice Cultures

Cultured brain slices were prepared following a protocol introduced by Stoppini and co‐workers (Stoppini et al. 1991) and as described in detail recently (Lerchundi et al. 2019; Gee et al. 2017). Briefly, acute brain slices were generated from hippocampi and forebrain of Balb/C mice (bred and raised at the Animal Care and Use Facility of the Heinrich Heine University Düsseldorf, postnatal day (P) 6–8, both sexes). Slices were quickly transferred under sterile conditions onto Biopore membranes (Millicell standing insert, Merck Millipore, Burlington, USA; Cat# PICM0RG50). They were then maintained in an incubator at 36.5°C for 10–21 days at an interface between humidified carbogen (95% O_2_/5% CO_2_ [Messer Industriegase, Bad Soden, Germany; CAS# 124‐38‐9]) and organotypic culture medium composed of MEM (minimum essential medium; Sigma‐Aldrich, cat. no. M7278), 20% heat‐inactivated horse serum (Origin: Brazil; Life Technologies GmbH, Darmstadt, Germany; Cat# 26050088), 1 mM L‐glutamine (Life Technologies GmbH, Darmstadt, Germany; Cat# 25030‐024), 0.01 mg/mL insulin (Sigma‐Aldrich, Taufkirchen, Germany; Cat# I6634), 14.5 mM NaCl (Carl Roth, Karlsruhe, Germany; Cat# 3957; CAS# 764714‐5), 2 mM MgSO_4_ (Life Technologies GmbH, Darmstadt, Germany; Cat# 213115000), 1.44 mM CaCl_2_ (FLUKA Honeywell; CAS# 10035‐04‐8), 0.00125% ascorbic acid (Carl Roth, Karlsruhe, Germany; Cat# 2A760‐P.A., 50‐81‐7; CAS# 50‐81‐7) and 13 mM D‐glucose (Caelo, Bonn, Germany; Cat# 2580; CAS 14431‐43‐7). The medium was exchanged every 3 days. The recipes and protocols used here (Gee et al. 2017) ensure healthy and viable slices that can be maintained for weeks without requiring the use of antibiotics or antimycotics. While (to the best of our knowledge) secondary diabetic complications have not been reported when using this protocol, we may note that the glucose concentration in the culturing medium is significantly higher than in the brain. At the same time, we may also note that imaging experiments were performed in saline containing 5 mM glucose (see below).

### 2.4 Brain Sections

For studies of brain tissue sections, three adult (10–12 weeks, both sexes) C57BL/6 mice were killed by an overdose of isoflurane (ca. 2.5–3.0 Vol‐% in air) and perfused through the ascending aorta with a brief rinse of 0.9% saline followed by fixative (4% paraformaldehyde in 0.1 M PB; pH 7.3) for 15 min. The brains were taken out and immersion‐fixed in the same fixative to complete an overall fixation time of 2 h, and rinsed several times in PB. Cryostat sections (12–14 μm) and 40 μm vibratome sections were prepared.

### 2.5 Immunocytochemistry and Microscopy

Immunostaining of primary astrocytes was carried out by sequential incubation in Triton‐X 100 (0.2%, 3 min; Sigma‐Aldrich, cat. no. X100), normal horse serum (NHS, Vector Laboratories, Burlingame, CA, USA, cat. no. S2000; 10%, 30 min), primary antibody(ies) solution (in 1% NHS, overnight, 4°C), secondary antibody(ies) solution (1 h, RT), and if required streptavidin‐CY3 (1 h, RT). Antibodies and concentrations are listed in Table 1. The coverslips were placed on a drop of mounting medium. All reagents were dissolved in PB. The same protocol was used for staining of brain tissue, using either free‐floating vibratome sections or slide‐mounted cryostat sections. Vibratome sections were not pre‐treated with Triton‐X 100. Immunocytochemical controls were carried out in parallel with double stainings, by omission of either one of the two primary antibodies. The controls were negative, demonstrating that there is no spurious signal owing to the detection system or filter bleed‐through.

**TABLE 1:** List of immunochemicals used.

Antibody target	Host	Supplier, Cat#, RRID	Conc.
Primary antibodies			
GFAP‐Alexa 647	Mouse	Cell Signaling Technology, Danvers, MA, USA, cat. no. 3657, RRID:AB_10693037	1:100
PDP1	Rabbit	MyBioSource, San Diego, CA, USA, cat. no. MBS2521181, RRID:AB_2889401	1:100
PKCδ	Rabbit	Cell Signaling Technology, cat. no. 9616, RRID:AB_10949973	1:50
COX IV	Mouse	Cell Signaling Technology, cat. no. 11967, RRID:AB_2797784	1:500
PDH	Mouse	Abcam, Cambridge, UK, cat. no. ab110330, RRID:AB_10858459	1:1000
Phospho‐Ser^293^‐PDH‐E1α	Rabbit	Millipore (Merck), Darmstadt, Germany, cat. no. AP1062, RRID:AB_10616069	1:500
Secondary antibodies and further reagents			
Biotinylated anti‐rabbit	Goat	Vector Laboratories, Burlingame, CA, USA, cat. no. BA‐1000, RRID:AB_2313606	1:217
Anti‐mouse‐Alexa 488	Donkey	Jackson Immunoresearch Laboratories, PA, USA, cat. no. 715‐545‐151, RRID:AB_2341099	1:100
Anti‐rabbit‐CY3	Donkey	Jackson Immunoresearch Laboratories, PA, USA, cat. no. 711‐165‐152, RRID:AB_2307443	1:1000
Streptavidin‐CY3		Vector Laboratories, cat. no. SA‐1300	1:1000

Images were acquired using an epifluorescence microscope (Zeiss) equipped with a 100×, 1.3 N.A. oil immersion objective and a 2048 × 2048 pixel monochrome camera (Spot Insight 4, Diagnostic Instruments, Sterling Heights, Michigan, USA) with 7.45 μm square pixels and 14 bit sampling depth (CCD chip type KAI 4021). Channel overlay was done at the step of image acquisition using the software Spot 5.0 Advanced (SPOT Imaging Solutions, Diagnostic Instruments), at the same time correcting for chromatic aberration (Anlauf and Derouiche 2009).

### 2.6 Image Analysis

**FIGURE 1:**
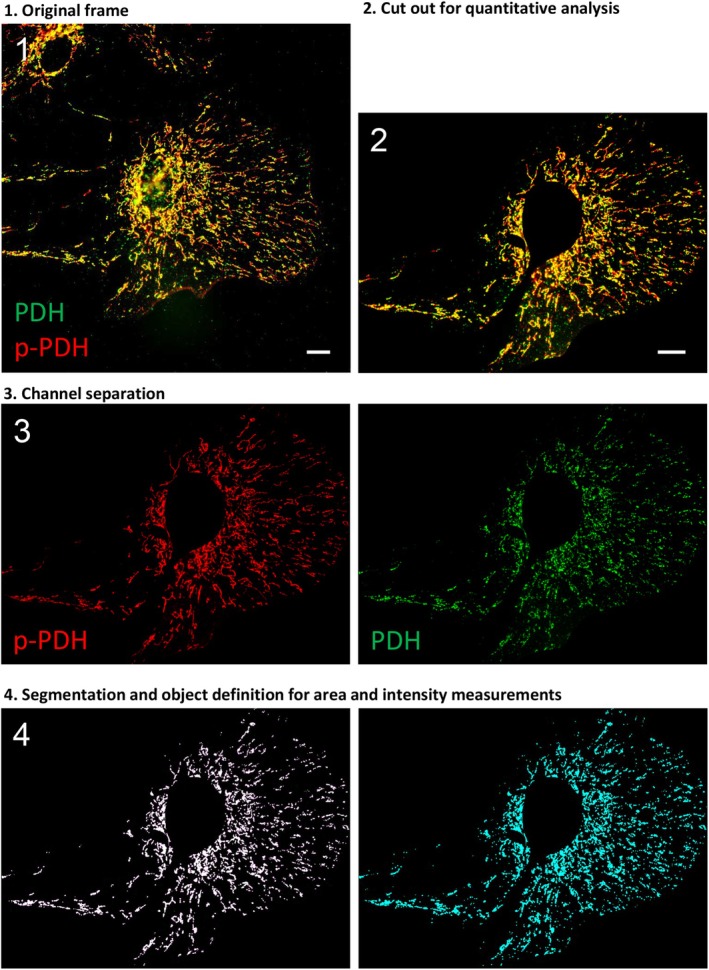
Quantitation of PDH‐phosphorylation. (1) The original frame of the double‐labeled cell shown in Figure 6A is cut out and trimmed (2) to exclude morphological features prone to producing erroneous measurements. (3) Fluorescence channels are separated; each channel is thresholded and segmented to result in numerous objects (4), as the basis for measurements of fluorescence signal intensity and area. See text. Scales: 10 μm (in 1, and in 2 for 2–4).

Quantification of PKCδ translocation to mitochondria, mitochondrial protein localization, and PDH phosphorylation were performed by object‐oriented image analysis using Volocity software (Version 6.3, Quorum Technologies, Puslinch, Ontario, Canada). In image acquisition and analysis, identical microscopy and exposure parameters and thresholds were applied to all frames to be included in a given quantitation. For presentation of side‐by‐side images comparisons, histogram operations were identical. Only linear procedures were used (threshold, cutoff). In PKCδ labelling, background threshold was set to cut off the diffuse signal thus allowing for definition of the faint puncta as discrete objects. The parameter “intensity density ratio” (IDR) developed previously (Farid and Derouiche 2019) serves as a quantitative measure of PKCδ translocation to the mitochondrial compartment. Briefly, the ratio of integrated fluorescence intensity values of mitochondrial vs. non‐mitochondrial PKCδ signal was calculated per cell and normalized to the integrated area of PKCδ^+^ objects (Farid and Derouiche 2019). The degree of PDH phosphorylation was quantitated in cells double‐stained for pyruvate dehydrogenase E1‐alpha subunit (PDH) and phospho‐Ser^293^ PDH (p‐PDH). The p‐PDH signal in a given astrocyte was integrated and divided by the cumulated area of the PDH signal, which appears to label complete mitochondria. The results were very similar when p‐PDH signal integration was based on intensity or area values. Cell regions particularly dense in mitochondria, such as the immediate perinuclear cytoplasm, and slender and filiform processes were cut out and excluded from the cell, focusing on its lamellar portions that have little optical overlay of mitochondria (Figure 1).

### 2.7 Pharmacological Testing, Media, and Controls

Compounds for pharmacological testing were L‐glutamate (Sigma‐Aldrich‐Aldrich, cat. no. G5889), 2‐Methyl‐6‐(phenylethynyl)pyridine hydrochloride (MPEP, Hello Bio, Bristol, UK, cat. no. HB0426), cPKC‐specific PKC inhibitor Gö6976 (Abcam, Cambridge, UK, cat. no. ab141413), and pan‐PKC‐inhibitor Gö6983 (Abcam, cat. no. ab144414). Test substances were prepared in HEPES‐buffered MEM (Sigma‐Aldrich, cat. no. M2414, 37°C) as stock solutions, and applied to achieve the following effective concentrations: MPEP (2 mM stock solution/100 μM effective concentration), Gö6976 (10 mM/1 μM), Gö6983 (10 mM/1 μM), and L‐glutamate (Sigma‐Aldrich, G5889; 100 μM, directly prepared). Combined application of L‐glutamate and either of the PKC inhibitors was preceded by pre‐incubation with the corresponding PKC inhibitor alone (37°C, 30 min (Li et al. 2014)). Testing of astrocyte cultures with pharmacological substances or glutamate was in Minimal Essential Medium (MEM; Sigma‐Aldrich, cat. no. M2414, glucose, 5.5 mM), since this appears to be the medium with the least amount of non‐essential substrates, in particular without glutamate, glutamine, lactate, or pyruvate.

### 2.8 Luminometric ATP Measurement

We adapted this assay from the similar ATP measurements in astrocytes by (Winkler et al. 2017). After incubation with pharmacological agents and subsequent cell lysis, ATP content was measured using a bioluminescence and luciferase‐based ATP determination kit (Molecular Probes/Invitrogen, cat. no. A22066), according to the manufacturer's instructions. Luminescence was recorded using a BMC Lumistar Microplate luminometer (BMG, Jena, Germany). We established an ATP standard curve starting at a concentration of 390 pM or 1.3 nM that was linear between 1.2 or 37 nM and 1000 nM (*R* ^2^ > 0.97). For experiments in 24‐well plates, two technical samples per time point and treatment were assayed since the variability was reasonably low. The manual procedure of substance application and cell lysis was standardized and monitored to keep the lag time in relation to the incubation times stated low. The mean lag time was 18 s. A multi‐channel pipette was used with 96‐well plates, rendering lag times largely irrelevant. Since the baseline values differed between time points, actual ATP concentrations [nM] were compared only within individual experiments. To integrate experiments, the results reported are percent values in relation to the corresponding time point baseline (control).

### 2.9 FRET‐Based ATP Imaging

Astrocytes in organotypic cultured slices were selectively transduced with an ATP‐sensor as reported recently (Lerchundi et al. 2019). To this end, 0.5 μL of a dilution of Adeno‐associated vector (AAV5/2; virus titer: 2.16 × 10^12^ viral genome/mL), diluted with DPBS was applied directly onto cultured slices, codifying the genetically‐encoded nanosensor ATeam1.03^YEMK^ (Imamura et al. 2009) under the transcriptional control of the human glial fibrillary acidic protein (GFAP) promoter fragment ABC1D‐GFAP (W. Frommer via Addgene, plasmid# 28004; RRID: Addgene 28 004). Transduced slices were maintained in the incubator at 36.5°C (95% O_2_/5% CO_2_ [Messer Industriegase GmbH, Bad Soden, Germany CAS# 124‐38‐9]) between 12 and 21 days until used in experiments. Imaging experiments were performed at 32°C–34°C.

For ATP imaging experiments, slices were transferred into an experimental bath constantly perfused with a saline composed of (in mM): 136 NaCl (Carl Roth, Karlsruhe, Germany; Cat# 3957; CAS# 764714‐5), 3 KCl (Carl Roth, Karlsruhe, Germany; Cat# 6781.1; CAS# 7447‐40‐7), 2 CaCl_2_ (Fluka/Honeywell; CAS# 10035‐04‐8), 1 MgCl_2_ (Carl Roth, Karlsruhe, Germany; Cat# 2189.1; CAS# 7791‐18‐6), 1.25 NaH_2_PO_4_ (Applichem, Darmstadt, Germany; Cat# 131965; CAS# 10049‐21‐5), 24 NaHCO_3_ (Life Technologies GmbH, Darmstadt, Germany; Cat# S4240/60) and 5 glucose (Caelo, Bonn, Germany; Cat# 2580; CAS# 14431‐43‐7), and bubbled with 95% O_2_ and 5% CO_2_ to obtain a pH of 7.35–7.4. The ATP sensor was excited at 434/17 nm using a lambda DG‐4 illumination (Nikon Instruments, Düsseldorf, Germany), coupled to an Eclipse FNI upright microscope (Nikon), which was equipped with an Achroplan 40× objective (water immersion, N.A. 0.8, Zeiss, Göttingen, Germany).

Ateam is a FRET‐based nanosensor. At low ATP levels, the fluorescence of the donor (enhanced Cyan Fluorescent Protein, eCFP; emission peaks at 475 nm) is high because the transfer of energy to the fluorescence acceptor (Venus; emission peaks at 527 nm) is low. Binding of ATP increases the energy transfer from donor to acceptor, which results in an increased fluorescence from Venus. The so‐called FRET ratio (i.e., the quotient of the fluorescence emission of Venus, divided by that of eCFP) can therefore serve as a reporter of changes in intracellular ATP levels. As excitation at 434 nm results in emission of fluorescence from both eCFP and Venus, Ateam fluorescence was split at 500 nm using a WVIEW GEMINI optic system (A12801‐01, Hamamatsu Photonics GmbH, Herrsching, Germany) and employing band‐pass filters at 483/32 and 542/27 (AHF Analysentechnik AG, Tübingen, Germany). Images were collected by a CMOS camera (Orca 4 LT Plus, Hamamatsu). Fluorescence was analyzed from regions of interest (ROIs) manually put around cell somata or processes of astrocytes employing the NIS‐Elements software (Nikon; RRID: SCR_014329). The FRET ratio (Venus/eCFP) was calculated after background correction and signals analyzed off‐line using OriginPro 9 Software (OriginLab Corporation, Northhampton, MA, USA; RRID: SCR_014212). Conversion of changes in the FRET ratio into changes in intracellular [ATP]_i_ was done based on an in situ calibration recently performed in our laboratory (Gerkau et al. 2019). Briefly, in this approach, slices were exposed to a calibration saline containing the saponin ß‐escin (Santa Cruz; Cat# sc491651; CAS# 111072‐93‐8), resulting in the formation of pores in the plasma membrane, allowing the free exchange of ATP between the extracellular saline and the cytosol. Changes in the FRET ratio following changes in the ATP concentration were recorded and fit with a Michaelis–Menten function; the resulting apparent *K*_D_ of the sensor was 2.6 mM (Gerkau et al. 2019); see also (Lerchundi et al. 2019).

### 2.10 Statistics

All IDR and PDH phosphorylation measurements were performed on cells of at least three independently prepared primary astrocyte cultures. Specimen identification was blinded for the experimental group prior to image acquisition and analysis; the selection of the microscopic field was done in the reference channel (COX IV, PDH, respectively). Statistical analysis was carried out using the programs BIAS 10.0 (Ackermann 2014) and SPSS Statistics 29 (IBM), with “*n*” = number of cells. Since the results based on primary astrocytes (Figures 4E, 6E,F, 7, 9B) report a novel effect using a novel quantitation method (protein translocation and phosphorylation by microscopy), we had no initial idea about effect size. In preliminary experiments, *n* = 20 was found to be by far sufficient, with preselected α = 0.05 and a statistical power (1‐ß) of 0.8. Power was calculated (using BIAS 10.0 (Ackermann 2014)) after significance testing (by Kruskal–Wallis), which was > 0.9 in all cases (Figures 4E, 6E,F, 7, 9B) corresponding to a large effect as assessed by eta (Pellerin and Magistretti 2012) or Cohen's d. For tests in primary astrocytes (Figures 4E, 6E,F, 7, 9B), the Kruskal–Wallis test was applied and followed by multiple Conover‐Iman comparisons (Bonferroni‐Holm‐corrected). Although not mandatory for this test, the data distributions were assessed and found to be normally distributed (Kolmogorov–Smirnov‐Lilliefors; *p* > 0.1 (BIAS 10.0 (Ackermann 2014))). The box plot diagrams for IDR, PDH phosphorylation, and luminometric ATP measurements indicate the median (line), interquartile range (IQR; box), and the whiskers indicate min/max values if within 1.5 × IQR above/below IQR; otherwise, the next value within 1.5 × IQR above/below IQR.

For FRET‐based ATP imaging in organotypic cultured slices, statistical analysis was performed using OriginPro 9.0. Prior to conducting parametric analyses (one‐way ANOVA), normality of the data distribution was assessed using the Shapiro–Wilk test (*α* = 0.05). Data sets showing *p* > 0.05 were accepted as normally distributed, permitting the use of one‐way ANOVA followed by Bonferroni post hoc analysis. “*n*” indicates the number of cells, and “N” indicates the number of individual experiments. Each experiment was performed on slices obtained from a total of five animals. In all analyses, *p* values < 0.05 were considered to indicate a significant difference. A power analysis was conducted using **GPower 3.1.9.3**. Assuming a significance level (*α* = 0.05) and a desired power of 0.80, the sample size employed (approximately 61 cells across the two conditions) was determined to be sufficient to detect a statistically significant effect, marked by “*”.

Statistical testing of microscopical cell measurements (Figures 4, 6, 7, 10) is based on the assumption that the unit of observation is the individual cell (*n* = 12–20, in each replicate experiment). As a note of caution, if the group means difference of the individual replicate experiment (*n* = 3) is taken as the unit of observation, test results are non‐significant.

## 3 Results

### 3.1 PKCδ and PDP1 Expression and Distribution in Astrocytes

**FIGURE 2:**
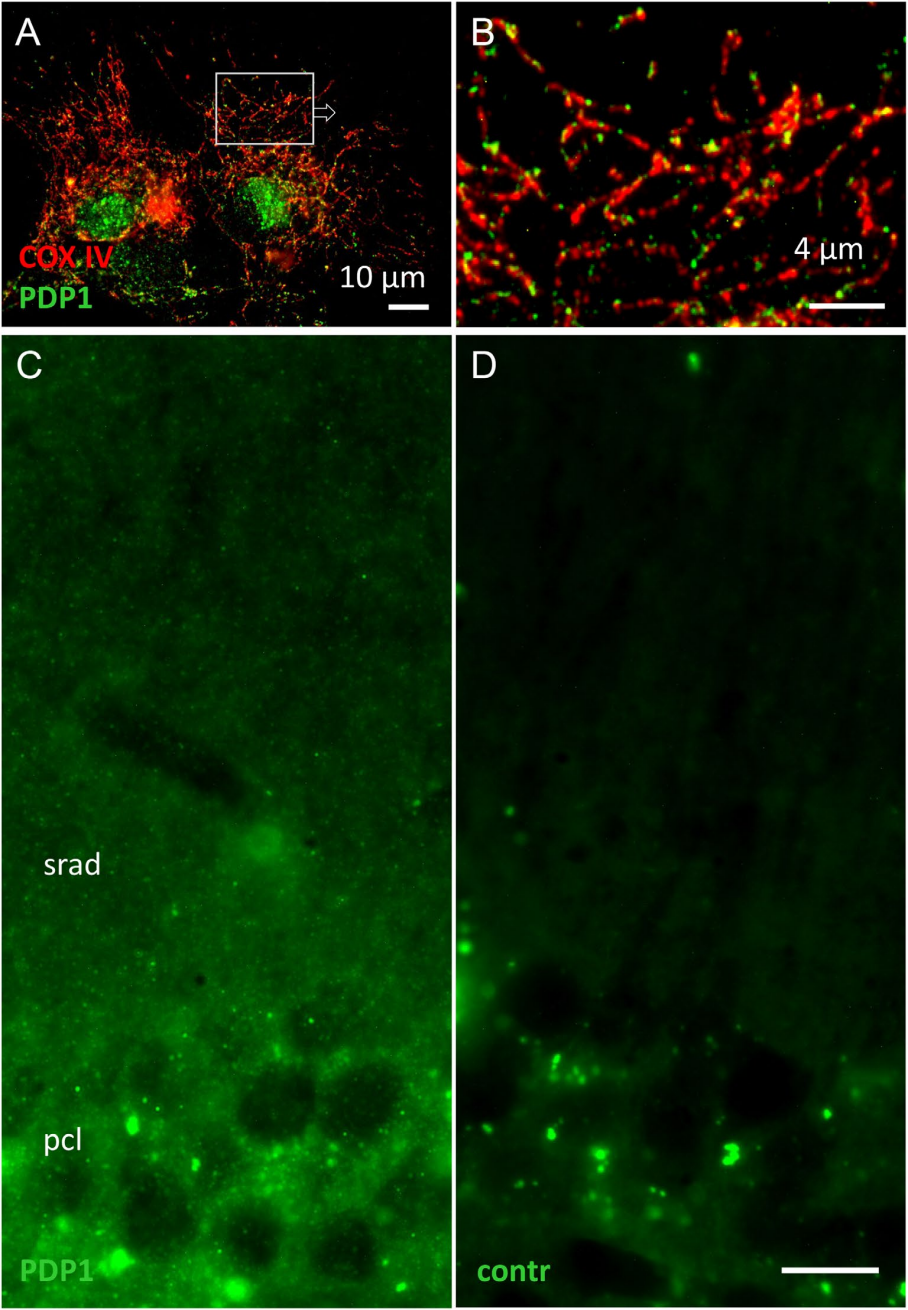
PDP1 is present in astrocytes. (A) In primary astrocytes, PDP1 immunostaining is mostly associated with mitochondria identified by COX IV staining. (B, magnification from frame in A) Whereas COX IV appears to reveal the complete mitochondrial shape, PDP1 staining is discretely punctate. (C) In brain sections, the PDP1 distribution is dense, homogeneously punctate, and above background fluorescence. This pattern is in line with staining in astrocytes, probably also in neurons and other cell types. (D) Control section, omission of primary ab. Mouse hippocampus, CA1; srad, stratum radiatum, pcl, pyramidal cell layer. Fluorescent granules in neuronal perikarya present also in control sections (D) are considered autofluorescent lipofuscin granules. Scales: 10 μm (A, D [for C and D]), 4 μm (B).

As a prerequisite for studies of a possible role of pyruvate dehydrogenase phosphatase 1 (PDP1) and PKCδ in astrocytic mitochondrial metabolism, we first investigated the presence and subcellular localization of these proteins in astrocytes. Immunocytochemical staining of cultured astrocytes reveals expression of PDP1, which is highly associated with mitochondria as identified by their marker protein cytochrome c oxidase IV (COX IV; Figure 2A,B). In the neuropil of the mouse hippocampus, very fine PDP1^+^ grains are distributed densely and homogeneously, in line with staining of mitochondria in astrocytes, not excluding other cell types (Figure 2C,D).

**FIGURE 3:**
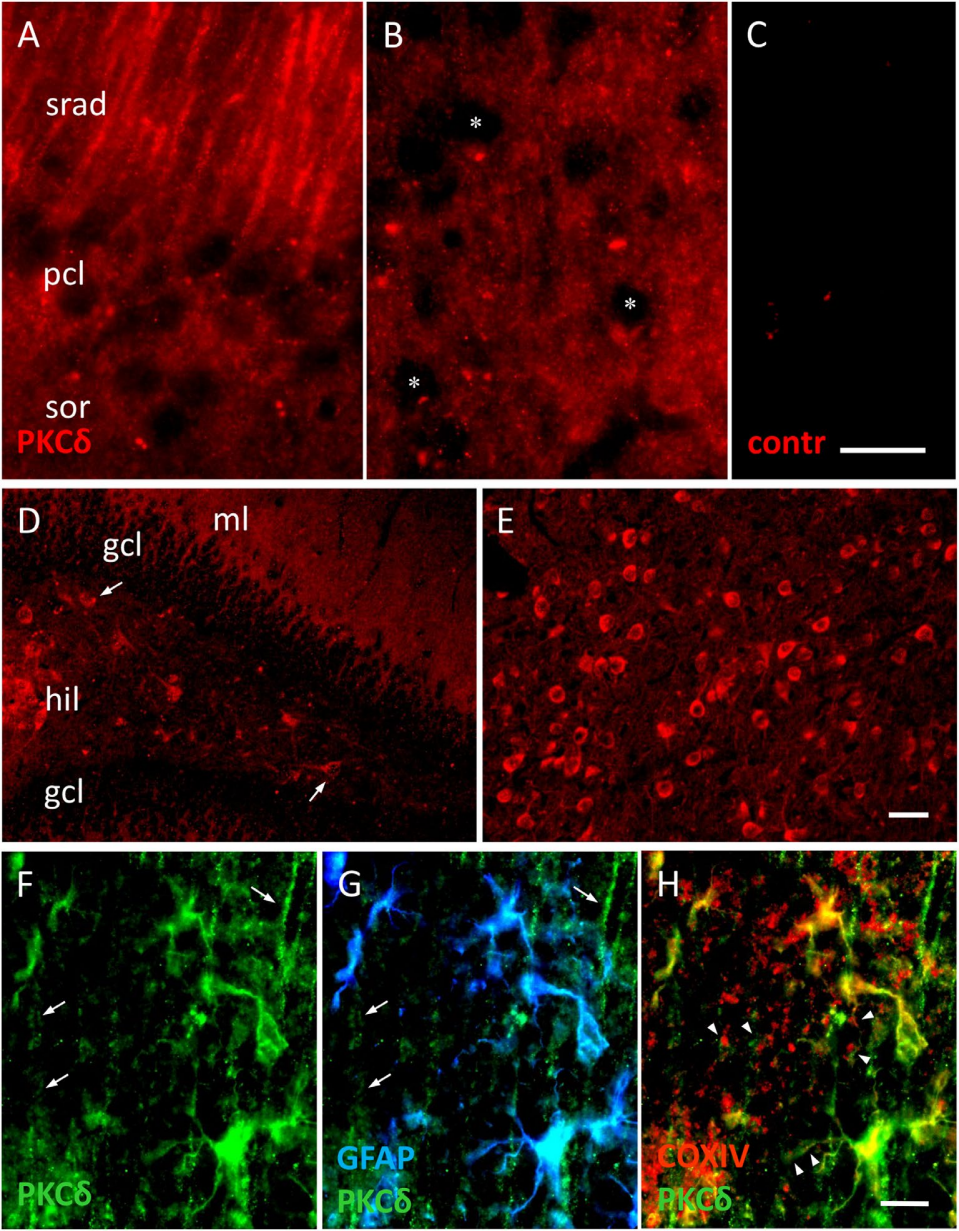
PKCδ is present in astrocytes and some neuron types. (A) Somata of hippocampal CA1 pyramidal cells and (B) cortical neurons (some indicated by asterisks) stand out negative against the diffuse neuropil staining, which is clearly above background (C, cortex, omission of primary ab). The dendrites in CA1, stratum radiatum (srad in A) are clearly labeled. (D) In fascia dentata, the molecular layer (mL) displays intense, diffuse label, while neurons in the granule cell layer (gcl) are negative. Fully labeled neurons are present in hilus (hil, some indicated by arrows) and (E) in thalamus. (F, G) Triple labeling. In addition to dendrites in srad of CA1 (arrows in F, G), PKCδ (green channel) also colocalizes with GFAP‐positive astrocyte stem processes (blue channel), and much of the diffuse PKCδ label may be in GFAP‐negative peripheral processes. (H) While PKCδ colocalizes with the full GFAP+ astrocytic shape (F), it may be focused around the COXIV+ astrocytic mitochondria (red channel, arrowheads in H). Pcl, pyramidal cell layer; sor, stratum oriens. Scales: 20 μm (C [for A–C]), 50 μm (E [for D and E]), 10 μm (H [for F–H]).

PKCδ is found abundantly in astrocytes in tissue sections of mouse forebrain, displaying a diffuse, even neuropil staining pattern with cut‐out perikarya of neurons, such as pyramidal cells and granule cells in the hippocampus, and most neuron types in cortex (Figure 3A–D). This is in line with previous reports (Brodie et al. 1998; Slepko et al. 1999). At the same time, PKCδ in neurons can be seen to be specifically localized to the dendrites of hippocampal pyramidal cells (Figure 3A), or completely label the somatodendritic compartment of neurons in hilus and thalamus (Figure 3D,E). Substantial PKCδ label is present in GFAP^+^ astrocytes, at higher magnification, both in stem processes and GFAP^−^ peripheral processes (Figure 3F,G). Of note, while PKCδ within astrocytes may accumulate around COX IV^+^ mitochondria, it is also distinctly non‐mitochondrial throughout astrocyte processes (compare Figure 3F,H).

**FIGURE 4:**
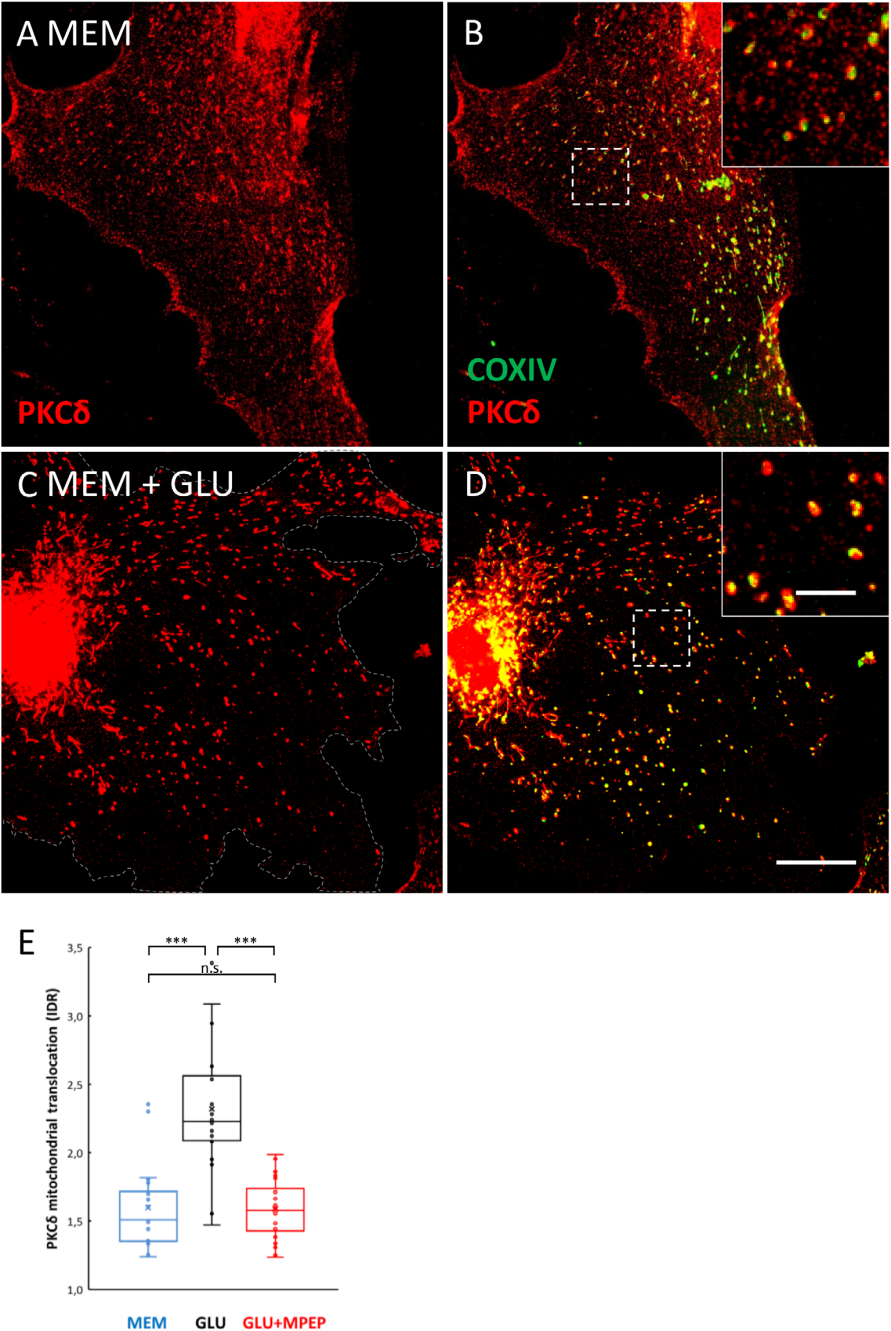
Glutamate induces mGlu5‐dependent mitochondrial translocation of PKCδ. PKCδ is localized in primary astrocytes and undergoes glutamate‐induced, mGlu5‐dependent mitochondrial translocation. (A) The pattern of subcellular PKCδ distribution in primary astrocytes is composite, with diffusely scattered small puncta and strands or larger particles. To minimize medium influence on cell metabolism, the culture medium was changed when applying the test solution, from DMEM (glucose, 5.5 mM) to MEM (glucose, 5.5 mM), which includes the least amount of non‐essential substrates, in particular no glutamate, glutamine, lactate, or pyruvate. (B) The strands or larger particles are identified as mitochondria by costaining with cytochrome oxidase IV. (C, D) Glutamate application (100 μm, 3 min) strongly diminishes the small punctate, extramitochondrial signal; compare insets in (B, D). (E) When quantitated by IDR (see text), mitochondrial translocation was highly significant. In the simultaneous presence of the specific mGlu5 antagonist MPEP (100 μM) this glutamate effect was completely blocked. Number of cells analyzed for each treatment *n* = 20; Number of independent replicates *N* > 3. Kruskal–Wallis (df = 2, H = 25.819, *p* = 0.000003), ***p < 0.000001; ns, non‐significant. Box plot labels: x = mean; line within box = median; whiskers = min/max if within 1.5 × IQR above/below IQR; else outlier dots and next value within 1.5 × IQR above/below IQR–Dashed line in (C) indicates cell boundary. Scales: 20 μm (in D, for A–D]), 5 μm (in insets).

**FIGURE 5:**
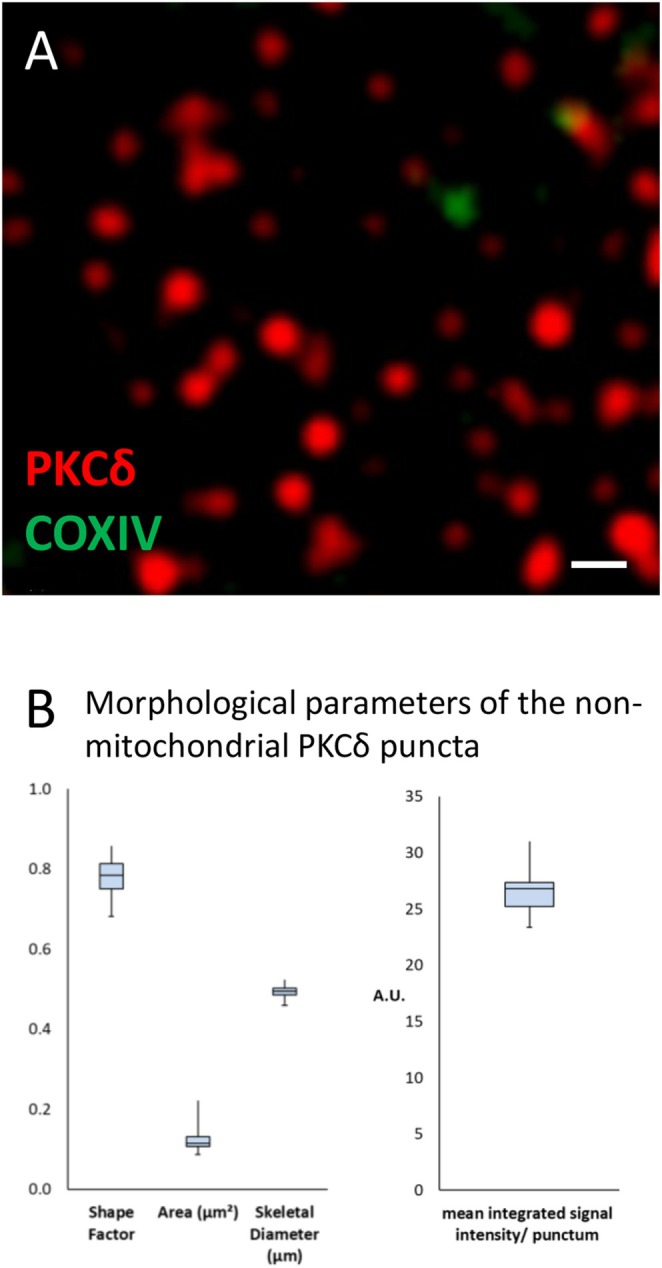
Non‐mitochondrial PKCδ puncta are highly uniform structures. (A) Note that the puncta at high magnification are mostly circular, varying in size by approximately 2×. (B) This is reflected by the narrow distributions of morphological parameters for roundedness (shape factor, 1 = circle), size (area, skeletal diameter), and mean integrated signal intensity/punctum. Whiskers indicate min/max values, all based on *n* = 935 puncta from *N* = 10 astrocytes from different cell culture preparations. Scale in (A): 500 nm.

Mitochondrial and non‐mitochondrial PKCδ localization is much more conspicuous in primary cultures of forebrain astrocytes. As expected, PKCδ^+^ label in the COX IV^+^ structures is large and intense, possibly outlining mitochondria (Figure 4A,B), which may be spherical in astrocytes (Derouiche et al. 2015). Surprisingly, non‐mitochondrial PKCδ is not cloudy throughout the cell but organized in discrete, evenly distributed puncta which are highly homogeneous in intensity, size, and shape (Figure 5). They might thus represent unitary, membrane‐bound PKCδ‐containing protein complexes involved in signaling (Uchino et al. 2004).

### 3.2 Glutamate Induces mGlu5‐Dependent Mitochondrial Translocation of PKCδ

In insulin‐stimulated hepatocytes, PKCδ translocates to mitochondria (Caruso et al. 2001). To test whether PKCδ similarly translocates to mitochondria in glutamate‐stimulated astrocytes, we applied glutamate to primary astrocytes. We initially chose a 3 min glutamate stimulus since intracellular ATP concentration [ATP]_i_ was found to drop dramatically after this duration (Winkler et al. 2017). Before substance application, primary astrocytes were changed from DMEM and adapted for 30 min to a medium devoid of glutamate, lactate, and pyruvate (MEM). Mitochondrial translocation was quantified by the parameter “intensity density ratio” (IDR), that is, the relation of mitochondrial vs. non‐mitochondrial PKCδ (on a per cell basis (Farid and Derouiche 2019)). Glutamate application (100 μM, 3 min) markedly reduces the punctate, non‐mitochondrial PKCδ, with a concomitant rise in apparent PKCδ intensity of the mitochondria (Figure 4C,D). This is reflected by an increase of the mean IDR value by 41.6% (Figure 4E). The punctate, scattered pattern of non‐mitochondrial PKCδ found in unstimulated cells transforms to a signal predominantly colocalizing with COX IV^+^ structures (Figure 4C,D), which we consider to reflect mitochondrial translocation of PKCδ.

Since mGlu5 is one of the two astrocytic mGluRs, and PKCδ can be activated by mGlu5 stimulation in mGlu5‐transfected cells (Uchino et al. 2004), we tested whether the PKCδ translocation observed here can be mediated by mGlu5. The glutamate‐induced IDR increase is largely abolished by simultaneous application of the specific mGlu5 antagonist 2‐Methyl‐6‐(phenylethynyl)pyridine hydrochloride (MPEP; 100 μM, Figure 4E). This suggests that mGlu5 stimulation by extracellular glutamate leads to mitochondrial translocation of PKCδ in astrocytes.

The presence of mGlu5 in mature astrocytes has been questioned (Sun et al. 2013). It can, however, be detected at low intensity in mature astrocytes (Panatier and Robitaille 2016), where it is systematically localized in the PAPs by silver‐intensified immunostaining (rat, adult; hamster, 3 months (Lavialle et al. 2011)). Also, the PAPs, which are easily torn off by tissue dissociation or fluorescence‐activated cell sorting procedures, contain a considerable amount of glial mRNA, which might explain the finding of undetectable mGlu5 mRNA (mouse, adult (Sun et al. 2013)).

### 3.3 Glutamate Induces mGlu5‐Dependent PDH Activation

**FIGURE 6:**
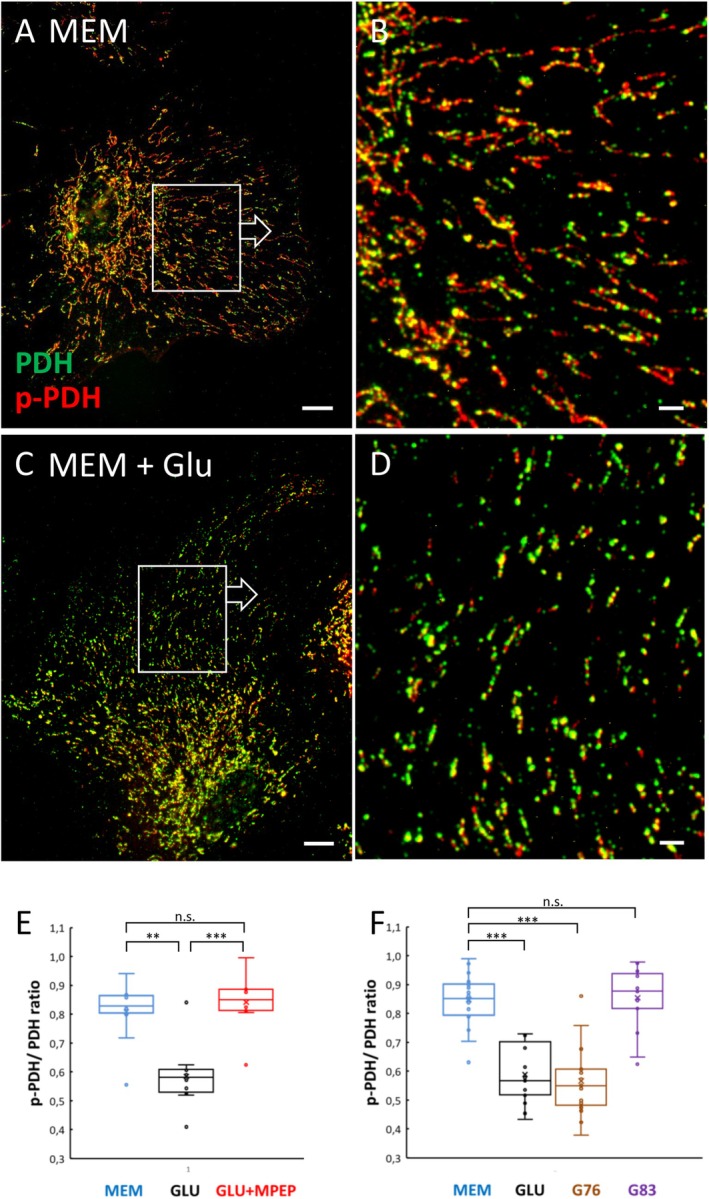
Glutamate induces mGlu5‐dependent dephosphorylation of PDH. (A and B, magnified as indicated) In control astrocytes (in MEM), mitochondria display a punctate staining with anti‐pyruvate dehydrogenase E1‐alpha subunit (PDH), but appear intensely and completely labeled by phospho‐Ser^293^ PDH (p‐PDH). (C and D, magnified as indicated) Medium application of glutamate (100 μM, 3 min) reduces the degree of p‐PDH label. For object‐oriented quantitation, the p‐PDH signal was related to that of PDH (p‐PDH/PDH ratio), either based on area or intensity (Figure 1). For the exemplary cells shown, the ratios are 0.828 (A, B) and 0.520 (C, D) based on area, and 1.113 and 0.569, respectively, based on intensity. (E) Mean, area‐based p‐PDH/PDH ratio values after simultaneous application of glutamate and MPEP (100 μM; mean = 0.842) are comparable to those with medium (MEM) alone (0.809). The glutamate effect (GLU; 0.585) is different from that of MEM (0.809) and glutamate plus MPEP (MPEP; 0.842; Kruskal–Wallis, df = 2, H = 13.737, *p* = 0.00104). PDH activation depends on PKCδ activity. (F) The significant decrease in PDH phosphorylation after glutamate alone (GLU; mean p‐PDH/PDH ratio = 0.589) remains unchanged when glutamate is simultaneously applied with Gö6976 (G76; 0.567), an inhibitor of the family of cPKCs not affecting PKCδ. The glutamate‐induced decrease in PDH phosphorylation is fully counteracted in co‐application of glutamate with Gö6983 (G83; 0.855), a broad inhibitor of cPKC isoforms as well as most nPKCs including PKCδ, comparable to the control (MEM, 0.849). To minimize medium influence on cell metabolism, the culture medium was changed when applying the test solution, from DMEM (glucose, 5.5 mM) to MEM (glucose, 5.5 mM), which includes the least amount of non‐essential substrates, in particular no glutamate, glutamine, lactate, or pyruvate. Scales: 10 μm (A, C), 2 μm (B, D). Number of cells analyzed for each treatment, in an individual experimental run *n* = 10 (E), *n* = 15–20 (F), number of experimental runs from independent cell culture preparations *N* = 3. Testing by Kruskal‐Wallis and multiple Conover‐Iman comparisons (Bonferroni‐Holm‐corrected), for (E) (df = 2, H = 13.737, *p* = 0.00104), **p < 0.002; ***p < 0.0003; n.s.—non‐significant; for (F) (df = 3, H = 40.020, *p* < 0.0001), ***p < 0.00001. Data for (E) are not normally, for (F) normally distributed (Kolmogorov‐Smirnov‐Lilliefors). Box plot labels: x = mean; line within box = median; whiskers = min/max if within 1.5 × IQR above/below IQR; else outlier dots and next value within 1.5 × IQR above/below IQR.

We next asked whether PKCδ when translocated to mitochondria, also mediates an increase in mitochondrial oxidative metabolism. PKCδ might be an activator of PDP1 and thus indirectly activate PDH. PDH, the key enzyme of the pyruvate dehydrogenase complex (PDC), irreversibly catalyzes pyruvate entry into the TCA cycle, thus enforcing an increase in mitochondrial oxidative metabolism and oxidative phosphorylation. The crucial regulatory site of PDH is Ser^293^ (Patel and Korotchkina 2006; Yeaman et al. 1978). Its dephosphorylation leads to activation, a process mediated in astrocytes by PDP1 (see above and (Halim et al. 2010)), which in turn requires PDP1 phosphorylation. We tested the possibility of glutamate‐induced changes in the phosphorylation state of Ser^293^, the target relevant for shifting both PDH activity and the metabolic profile of astrocytes. Before substance application, primary astrocytes were changed from DMEM and adapted for 30 min to a medium devoid of glutamate, lactate, and pyruvate (MEM), and afterwards fixed and double immunolabeled for PDH and pSer^293^‐PDH. Measurements of the pSer^293^‐PDH/PDH ratio as the test parameter are very similar when based either on intensity or area (Figure 1). Glutamate application (100 μM, 3 min) prior to fixation leads to a significant reduction of this ratio by 28.8% (area) or 28.3% (intensity), and this Ser^293^‐PDH dephosphorylation can be completely prevented by co‐application of MPEP (100 μM, Figure 6). This indicates that glutamate can indirectly lead to dephosphorylation and activation of PDH in astrocytes and that this effect is not mediated by intracellular, metabolic effects of glutamate but by its extracellular binding to mGlu5.

### 3.4 PDH Activation Depends on the Kinase Activity of PKCδ

**FIGURE 7:**
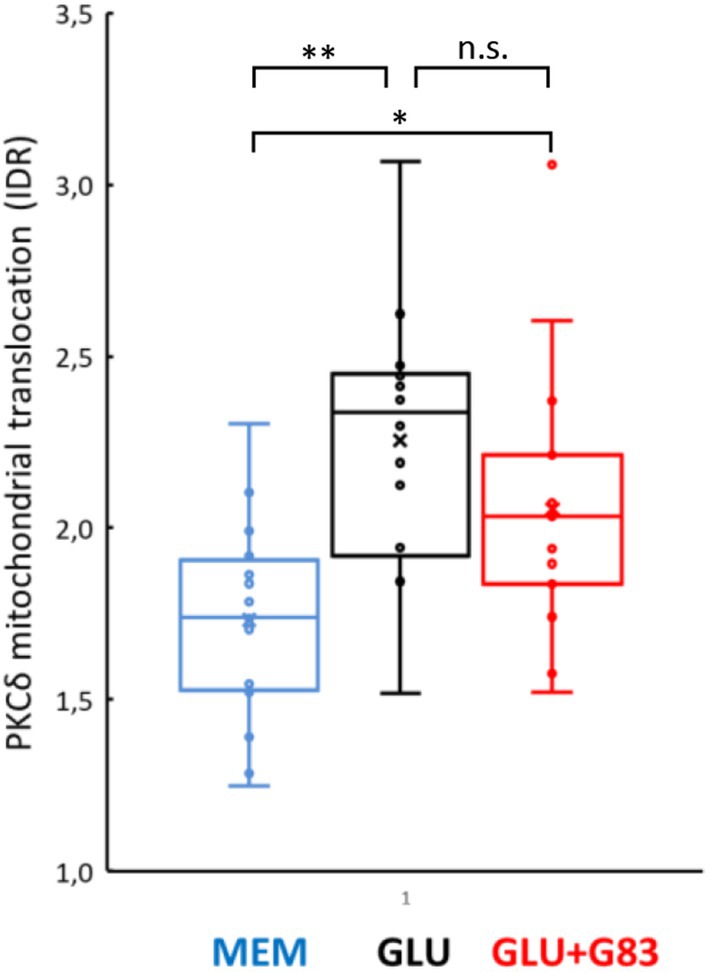
Gö6983 does not affect glutamate‐induced mitochondrial translocation of PKCδ. Mitochondrial translocation of PKCδ as quantitated by IDR (see text) is increased over control (1.73 ± SD 0.29) after application of glutamate (100 μM, 3 min, compare Figure 4; 2.26 ± 0.39), and this glutamate effect is not blocked by co‐application of Gö6983 (1 μM; 2.06 ± 0.39). To minimize medium influence on cell metabolism, the culture medium was changed when applying the test solution, from DMEM (glucose, 5.5 mM) to MEM (glucose, 5.5 mM), which includes the least amount of non‐essential substrates, in particular no glutamate, glutamine, lactate, or pyruvate. MEM, Minimal Essential Medium (control); Glu, glutamate; Glu+G83, glutamate and Gö6983. Minimal number of primary astrocytes analyzed for each condition, within one cell culture preparation, *n* = 12; two independent cell culture preparations i.e., experimental runs. Testing by Kruskal–Wallis (df = 2, H = 14.192, *p* = 0.000828) and multiple Conover‐Iman comparisons (Bonferroni‐Holm‐corrected), *p < 0.05; **p < 0.001, n.s.—non‐significant. Data are normally distributed (Kolmogorov–Smirnov‐Lilliefors). Box plot labels: x = mean; line within box = median; whiskers = min/max if within 1.5 × IQR above/below IQR; else outlier dots and next value within 1.5 × IQR above/below IQR.

**FIGURE 8:**
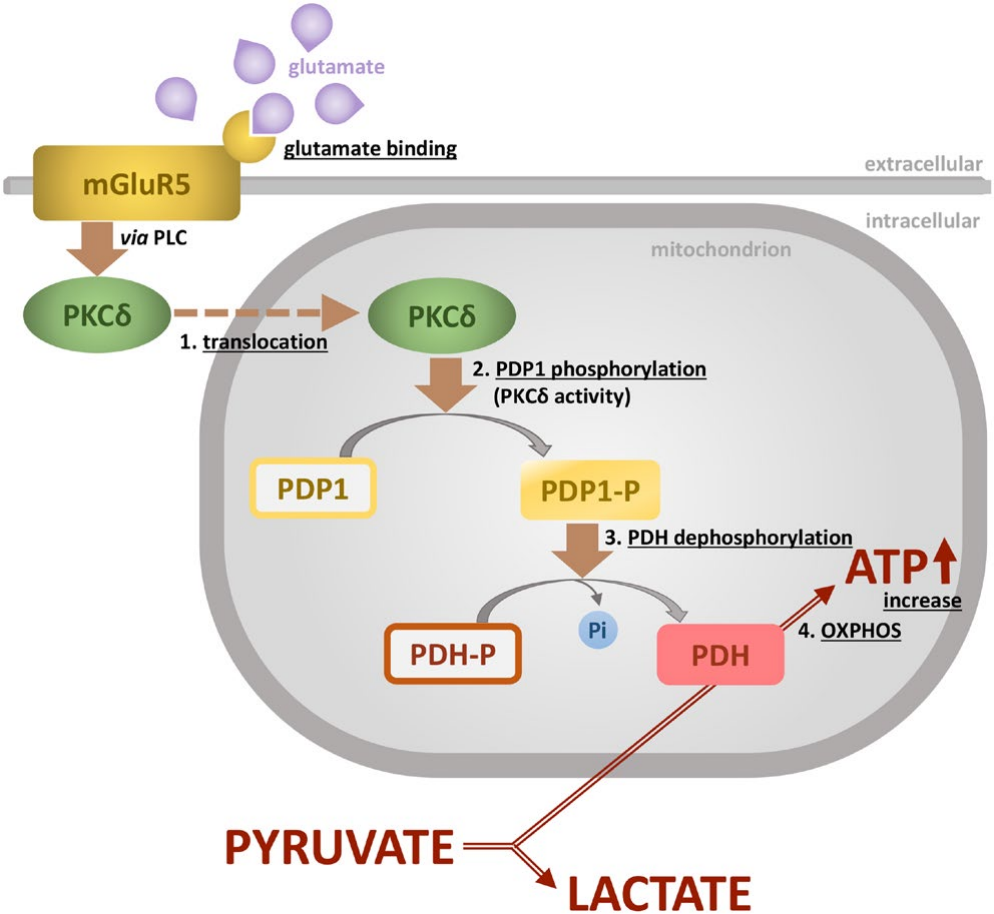
Model of glutamate‐induced regulation of oxidative phosphorylation. Pyruvate can be metabolized to lactate, by cytosolic LDH. The key step for channeling pyruvate to mitochondrial degradation resulting in high ATP yield, is accomplished by PDH. The most effective mechanism of its activation is dephosphorylation by PDP1. In the model proposed here, PDP1 in astrocytes is activated by PKCδ. Activation and mitochondrial translocation of PKCδ follows after glutamate stimulation of mGlu5, an effect probably mediated by PLC. The steps underlined have been shown to be stimulated by glutamate and to depend on mGlu5 activation.mGlu5, metabotropic glutamate receptor 5; PDH, pyruvate dehydrogenase; PDP1, pyruvate dehydrogenase phosphatase 1; PLC, phospholipase C; PKCδ, protein kinase C delta.

Although mitochondrial translocation of PKCδ and PDH dephosphorylation may be causally linked (Caruso et al. 2001), alternative pathways might involve additional protein kinases activated by mGlu5. We further asked whether PKCδ kinase activity could mediate PDH activation. We tested whether Ser^293^‐PDH dephosphorylation is indirectly induced by PKCδ kinase activity (via PDP1) by co‐applying PKC kinase activity inhibitors with glutamate (100 μM, 3 min) to cultured astrocytes. Given the lack of a subtype‐selective PKC inhibitor specific for PKCδ, we logically combined Gö6976, an inhibitor of the family of cPKCs not affecting PKCδ (Gschwendt et al. 1996), and Gö6983, a broad inhibitor of cPKC isoforms as well as most nPKCs including PKCδ (Gschwendt et al. 1996). As shown biochemically, Gö6983 also inhibits the kinase activity of PKCδ without affecting its translocation ability (Gschwendt et al. 1996), in line with the absence of morphological translocation in our IDR paradigm (Figure 7). While Gö6976 (1 μM), which does not target PKCδ, does not affect the glutamate‐induced PDH phosphorylation, this phosphorylation is completely blocked by Gö6983 (1 μM), a broad PKC inhibitor including PKCδ (Figure 6F). Although not strictly excluding the possible involvement of other protein kinases, this observation is in line with our assumption that the glutamate‐induced and mGlu5‐dependent PDH activation is mediated by the kinase activity of PKCδ. Taken together, the observations strongly suggest that mGlu5‐mediated mitochondrial translocation of PKCδ in astrocytes indirectly induces PDH dephosphorylation via activation of PDP1 (Figure 8).

### 3.5 Influence of mGlu5 on ATP Regeneration after Glutamate Exposure in Primary Astrocytes

**FIGURE 9:**
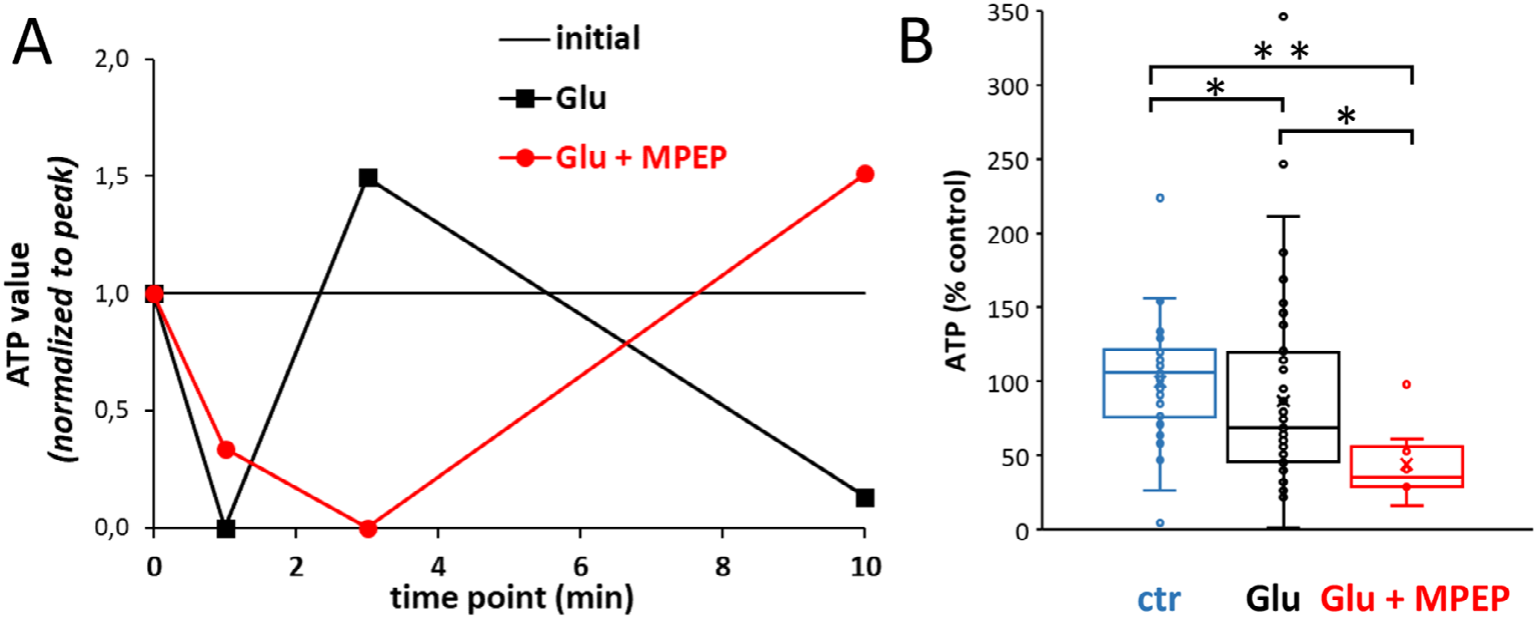
Influence of mGlu5 on ATP regeneration after glutamate exposure in primary astrocytes. (A) Initial time course of ATP values after application at time point 0 min., of glutamate (100 μM, black) or glutamate plus MPEP (100 μM each, red). ATP values were first calculated as percent of control (initial value, thin black line) and then normalized to the range between initial value (1) and the minimum of either trace (0). (B) Comparison of the drops in ATP (percent of control) after substance application, which was deepest after 1 min for glutamate (mean = 86, 7; number of wells measured = 52) and after 3 min for glutamate plus MPEP (44, 1; *n* = 10). Testing by Kruskal‐Wallis (df = 2, H = 15.941, *p* = 0.000346) and multiple Conover‐Iman comparisons (Bonferroni‐Holm‐corrected), *p < 0.05; **p < 0.001. To minimize medium influence on cell metabolism, the culture medium was changed 30 min before testing, from DMEM (glucose, 5.5 mM) to MEM (glucose, 5.5 mM), which includes the least amount of non‐essential substrates, in particular no glutamate, glutamine, lactate, or pyruvate.

With these signaling steps suggested so far, mGlu5‐mediated PDH activation would irreversibly channel pyruvate to oxidative phosphorylation in the mitochondrion, thus enhancing ATP generation (Patel et al. 2014). We further addressed this proposed mechanism by measuring astrocytic ATP production. We applied an ATP luminescence assay in astrocytes stimulated with glutamate for 1, 3, or 10 min, with or without mGlu5 inhibition, cultured and lysed in 24 or 96 well plates. Intracellular ATP in live cultured astrocytes has been previously studied at the single cell level (*n* = 8–700) by online time‐lapse microscopy using a genetically encoded fluorescent ATP sensor (Winkler et al. 2017). In a 3 h time series, with initial time points 10 and 35 min, these authors observed a steep, initial drop of [ATP]_i_ at 10 min after glutamate (100 μM) application, and recovery at 35 min (figures 2B, f in (Winkler et al. 2017)). To compare the pattern of drop and recovery in our experiments, [ATP]_i_ values were normalized to the maximal drop. The reduction of ATP values by glutamate (100 μM) is maximal already after 1 min, recovers after 3 min, and falls again by 10 min. In contrast, in the presence of MPEP (100 μM) the glutamate‐induced [ATP]_i_ drop continues until 3 min and recovers at 10 min (Figure 9A). The deepest glutamate‐induced drops of [ATP]_i_ values are 86.7% of control ±64.1 (SD, *n* = 52 wells) at 1 min, and 44.1% ± 23.53 at 3 min when glutamate is combined with MPEP (*n* = 10). The effects of glutamate alone and glutamate plus MPEP differ significantly (*p* < 0.05) from each other and from control (100% ± 37.14; *n* = 46) (Figure 9B).

Based on our chosen single time points of 1, 3, and 10 min, we cannot analyze the time course of [ATP]_i_ drop and initial recovery at higher temporal resolution. The glutamate‐induced drop at 10 min reported (Winkler et al. 2017) may correspond to the secondary drop after the initial 1 min drop and interim recovery, which we observe (Figure 9A) and which would be concealed by the low temporal resolution (10 min) of the experiments by Winkler et al. (Winkler et al. 2017). Of note, Winkler et al. (2017) also showed a remarkably high variability of [ATP]_i_ values. Thus, although mean [ATP]_i_ initially dropped after glutamate application in a concentration‐dependent manner, 10%–20% of the individual cells stayed level or even increased in relation to control (figure 2 B in (Winkler et al. 2017)), and at 35 min, the recovery led to overshoot above normal in 20% of cells (figure 2F in (Winkler et al. 2017)). In conclusion, our measurements show that in the presence of MPEP, the temporal sequence of the glutamate‐induced [ATP]_i_ drop and recovery is delayed, and the initial drop is deeper. This further supports the hypothesized role of mGlu5 in ATP generation (Figure 8).

### 3.6 mGlu5 Supports Regeneration of Astrocytic ATP after Glutamate Exposure in Organotypic Brain Slice Culture

**FIGURE 10:**
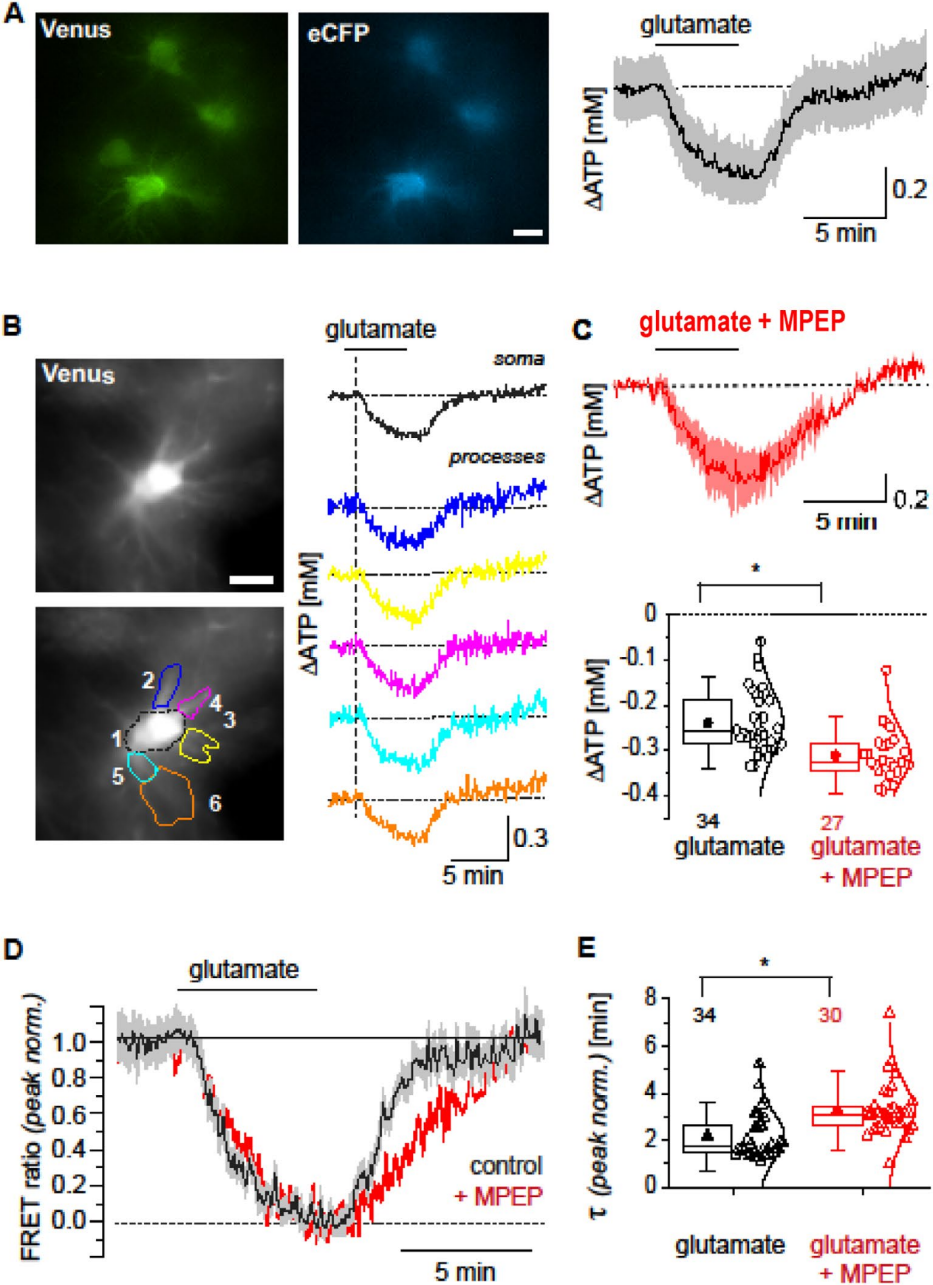
Glutamate‐induced changes in astrocytic [ATP] and role of mGlu5 in organotypic brain slices. (A) left and center: Images of Venus and eCFP fluorescence in astrocyte tissue slices transfected with ATeam1.03^YEMK^. Scale bar: 10 μm. Right: Change in astrocytic [ATP] in response to a 5 min exposure to 500 μM glutamate as detected with ATeam1.03^YEMK^. The black trace shows the mean; the gray traces represent single cells in one experiment. (B) Left: Image of the Venus fluorescence of an astrocyte expressing ATeam1.03^YEMK^ (top). Note that the contrast of the original image was increased to better visualize the fine cellular processes. Bottom: Selected regions of interest (ROI) from which ATeam1.03^YEMK^ fluorescence was collected as illustrated on the right. Scale bar: 10 μm. Right: Change in [ATP] in the individual ROIs in response to a 5 min exposure to 500 μM glutamate. C. Top: Change in astrocytic [ATP] in response to a 5 min exposure to 500 μM glutamate in the presence of MPEP. The dark red trace shows the mean; the light red traces represent single cells in one experiment. Bottom: Box chart depicting mean values (squares), IQR (box), median (line), SD (whiskers) and all single data points (open dots) of peak changes in astrocytic [ATP] induced by glutamate (black) and by glutamate in the presence of MPEP (red). The numbers above the boxes indicate the number of analyzed cells; *: *p* < 0.05 (one‐way ANOVA, *F*(1, 59) ≈5.10, *p*≈0.028; Bonferroni post hoc test). (D) peak‐normalized traces (same as in A and C, respectively), showing changes in the ATeam FRET‐ratio in response to a 5 min exposure to glutamate and in the presence (red) and absence (black) of MPEP. (E) Box chart depicting mean values (triangles), IQR (box), median (line), SD (whiskers), and all single data points (open triangles) of the time constant τ of the recovery from glutamate‐induced decreases (normalized) and in the presence (red) and absence (black) of MPEP. The numbers above the boxes indicate the number of analyzed cells; *: *p* < 0.05 (one‐way ANOVA, *F*(1, 62) ≈7.80, *p*≈0.007; Bonferroni post hoc test).

The involvement of mGlu5 on glutamate‐induced changes in astrocytic [ATP]_i_ was further studied in organotypic brain slice cultures. To analyze changes in [ATP]_i_ evoked by glutamate, astrocytes were selectively transfected with the sensor ATeam1.03^YEMK^ (Figure 10A). Imaging experiments were performed in the presence of tetrodotoxin (TTX, 0.5 μM) to block voltage‐gated sodium channels and of NBQX (2,3‐dihydroxy‐6‐nitro‐7‐sulfamoyl‐benzo[f]quinoxaline, 20 μM) and APV ((2R)‐amino‐5‐phosphonovaleric acid, 50 μM) to inhibit ionotropic glutamate receptors. After recording a baseline for at least 10 min, slices were then exposed to 500 μM glutamate for 5 min. Glutamate application results in a transient decrease in the [ATP]_i_ in astrocyte somata by 0.24 ± 0.07 mM (*n* = 34, *N* = 5) (Figure 10A,C). The peak amplitude and time course of glutamate‐induced changes in [ATP]_i_ are essentially indistinguishable between somata and cellular processes (*n* = 15, *N* = 4) (Figure 10B).

To study the involvement of mGlu5 in glutamate‐induced changes in astrocytic [ATP]_i_, slices were first perfused with the mGlu5 blocker MPEP (100 μM) for 15 min. In the continued presence of MPEP, the peak amplitude of the glutamate‐induced decrease in [ATP]_i_ is significantly larger (*p* < 0.05) compared to control, amounting to 0.31 ± 0.06 mM (*n* = 27, *N* = 5) (Figure 10C). In addition, we find that the recovery from glutamate‐induced decreases in [ATP]_i_ was slowed in the presence of MPEP. To directly compare the time course and kinetics of recovery to baseline [ATP]_i_ under both conditions, changes in [ATP]_i_ were peak‐normalized and the recovery phase fit monoexponentially (Figure 10D). While under control conditions, the time constant τ is 2.15 ± 0.97 min (*n* = 34, *N* = 5), it increases significantly to 3.33 ± 1.2 min (*n* = 30, *N* = 5) with MPEP (Figure 10E). In organotypic brain slice cultures, glutamate application with inhibition of mGlu5 thus leads to larger decreases and slowed recovery of astrocytic [ATP]_i_. The effect of mGlu5 inhibition on glutamate‐induced [ATP]_i_ drop and recovery is comparable in primary astrocytes (see above) and those in organotypic brain slice culture, possibly indicating a shared mechanism.

## 4 Discussion

### 4.1 An Astrocytic Signaling Pathway for Extracellular Glutamate to Regulate Oxidative Metabolic Capacity

We demonstrate by immunocytochemistry the presence of PKCδ and PDP1 in astrocytes in vitro and in mouse forebrain sections. In primary astrocytes, mitochondrial translocation of PKCδ and PDH dephosphorylation can be induced by glutamate, depend on mGlu5 activation but independent of glucose, lactate, or pyruvate. We also show that PDH activation through dephosphorylation depends on the kinase activity of PKCδ. In astrocytes in cell culture and in organotypic brain slice culture, mGlu5 activation supports regeneration of astrocytic ATP after glutamate exposure. This leads us to propose a mechanism in astrocytes where glutamate‐stimulated and mGlu5‐mediated mitochondrial translocation of PKCδ indirectly induces PDH dephosphorylation, via phosphorylation of PDP1, to finally increase mitochondrial ATP production, via oxidative phosphorylation (Figure 8). A similar metabolic effect in *Drosophila* has been suggested very recently where neuronal PKCδ intervenes in long‐term memory formation, involving dopamine receptor activation and PDH disinhibition (Comyn et al. 2024).

Checking the plausibility of such a mechanism, all components of the PDC including PDH are present in astrocytes (Halim et al. 2010). PDC activity is kept strongly inhibited in cultured astrocytes through PDH phosphorylation (Halim et al. 2010). In hepatocytes and skeletal muscle, PDH activation by PKCδ can be induced by insulin in a process where PKCδ translocates to the mitochondrion and activates PDP1/2 (Caruso et al. 2001). These findings (Caruso et al. 2001) form the basis for the hypothesis of our study. In astrocytes, PKCδ is cytosolic but mostly associated with organelles (Slepko et al. 1999); it is involved in regulating the expression or localization of key astrocytic proteins, such as GFAP, glutamine synthetase, GLT‐1, and aquaporin 4 (Brodie et al. 1998; Wang et al. 2017; Noël et al. 2021).

PDH activity is regulated mainly through the degree of phosphorylation of the PDH α‐subunit of E1 (E1α), which displays three serine residues as phosphorylation sites (Rardin et al. 2009), and is activated by dephosphorylation. Ser^293^, Ser^300^, and Ser^232^ (sites 1, 2, and 3) are modified by PDH‐kinases (PDK 1–4) and PDH‐phosphorylases (PDP 1, 2). In cultured astrocytes, the prevailing enzyme isoforms are PDK2 and PDP1, respectively (Halim et al. 2010); our immunocytochemical data in mouse brain confirm the Western blot data that PDP1 prevails in astrocytes (Halim et al. 2010). Site 1 is the one phosphorylated most rapidly and the one dephosphorylated most slowly, yet it contributes most strongly to inactivation and up to 97% to the increase in PDH activity (Patel and Korotchkina 2006; Yeaman et al. 1978). The anti‐phospho PDH antibody applied here specifically detects this site.

Furthermore, it has been shown previously that PKCδ can be activated by mGlu5 stimulation in transfected cells (HEK cells (Uchino et al. 2004)). It remains to be studied whether the presently observed punctate pattern of non‐mitochondrial PKCδ immunolabeling (Figures 4A and 5) might correspond to membrane signaling spots where mGlu5 and PKCδ are anchored (Uchino et al. 2004). When activated, PKCδ can translocate to mitochondria (Wu‐zhang et al. 2012) and translocation of PKCδ appears a prerequisite for affecting the mitochondrial enzymes of the TCA cycle, presumably via an on‐site signalosome complex. An exact molecular mechanism has been proposed with PKCδ activating the mitochondrion in a highly regulated, redox‐dependent manner, via a signalosome protein complex consisting of PKCδ, p66Shc, retinol, and cytochrome c (Acin‐Perez et al. 2010; Kim and Hammerling 2020). Our results suggest the additional requirement of PKCδ kinase activity for transducing signals from activated mGlu5 since Gö6983, which inhibits kinase activity but not translocation of PKCδ, counteracts PKCδ‐induced PDH activation (Figure 6F).

Regarding a mechanism linking PKCδ kinase activity to PDH regulation in astrocytes, PKCδ might in principle inhibit PDK2 (mouse embryonic fibroblasts (Acin‐Perez et al. 2010)) or activate PDP1 (hepatocyte and skeletal muscle (Caruso et al. 2001)). We currently demonstrate the presence of PDP1 in astrocytes in mouse brain sections (Figure 2C,D), and in extracts of cultured astrocytes, the addition of recombinant PDP considerably enhances PDH activity (Halim et al. 2010).

### 4.2 Possible Spatiotemporal Tuning of Glutamate‐Induced Metabolic Adaptation

Traditionally, there has been a debate as to whether astrocytic support of neurotransmission is primarily based on glycolysis or on mitochondrial ATP generation by oxidative phosphorylation (Pellerin and Magistretti 1994; Dienel et al. 2023; Dienel 2012; Fernandez‐Moncada et al. 2018; Magistretti and Chatton 2005). One argument against the latter was the frequently observed fine morphology of PAPs (< 100 nm (Reichenbach et al. 2010)), considered too small to accommodate mitochondria (Hertz et al. 2007; Stephen et al. 2014). However, morphological studies (Lovatt et al. 2007; Derouiche et al. 2015; Owens et al. 2015) showed that PAPs in fact contain numerous very small, often spherical mitochondria (200–400 nm) that had been overlooked before. Again, it was argued (Petit and Magistretti 2016) that PDH, the mitochondrial enzyme responsible for the entry of pyruvate into the TCA cycle, is saturated under basal metabolic conditions (Halim et al. 2010), meaning that when excess glucose is taken up by astrocytes, it cannot enter the TCA cycle, and lactate will be produced. However, PDH activity in astrocyte extracts can be released by adding PDP enzyme or inhibiting the inhibitory PDKs, which reduces lactate production (Halim et al. 2010). Also, the reserve capacity for increasing PDH activity is particularly high (> 300%) in cultured astrocytes, since their basal PDH activity levels are low (Halim et al. 2010).

Our observations suggest a new mechanism by which astrocytes can regulate the balance between their glycolytic and oxidative metabolism (Dienel 2013; Dienel et al. 2023; Hertz et al. 2007), namely the mitochondrial translocation of PKCδ which depends on mGlu5 stimulation. It appears that the plethora of astrocytic mitochondria may maintain a basal level of oxidative metabolism, with a considerable stand‐by potential for add‐on capacity that can be stimulated by extracellular glutamate.

Regulation of glial energy metabolism is important in the context of neuronal activity. Astrocytes change their gene expression patterns for important metabolic enzymes in response to increased nearby neuronal activity (Hasel et al. 2017). However, even in acute glial gene regulation by synaptic activity via mechanisms involving cAMP/PKA‐dependent CREB activation (Hasel et al. 2017), this process (i) requires hours and (ii) involves the metabolome of the whole cell. In contrast, the receptor‐mediated, mGlu5/PKCδ/PDH pathway proposed here may be operational in astrocytes to permit a fast adaptation to increased energy demand over short, acute phases (s–min). It is noteworthy here that even the PAPs in situ display mGlu5 (even selectively so (Lavialle et al. 2011)), PKCδ (Figure 3F–H), PDP1 (Figure 2C,D), and very small mitochondria (Lovatt et al. 2007; Derouiche et al. 2015; Owens et al. 2015). It is conceivable thus that the proposed acute up‐regulation of astrocytic oxidative metabolism via mGlu5/PKCδ may be locally restricted to glial microdomains (Grosche et al. 1999) consisting of PAPs and enclosing active synapses, without changing the metabolic state of other parts of the astrocyte territorium, the perinuclear region, or the whole cell. The integration of the proposed signaling mechanism into overall astrocyte and brain energy metabolism remains to be further studied.

### 4.3 The Findings in the Context of Glial Metabolism and Synapse Physiology

Several further points indirectly related to the data deserve mention. Thus, the astrocyte‐neuron‐lactate shuttle (ANLS) hypothesis (Pellerin and Magistretti 2012; Pellerin and Magistretti 1994) mentioned above cannot be reconciled with the glutamate‐induced increase in PDH activity reported here since the latter does not produce lactate. As pointed out by (Dienel 2017), the original observations leading to the ANLS hypothesis (Pellerin and Magistretti 1994) do not support the model at the level of stoichiometry. Similarly, in the ANLS model, lactate oxidation would require the match of glucose utilization and oxygen consumption, which is contradicted however by the finding of 50% and only 5% increase under somatosensory stimulation, respectively (Fox and Raichle 1986) (humans), and such mismatch has been observed in other examples of activation in animals and humans (reviewed in (Dienel 2019)). These arguments together with the presence of mitochondria in PAPs (Lovatt et al. 2007; Derouiche et al. 2015; Owens et al. 2015) and the high reserve capacity for increasing PDH activity (Halim et al. 2010) speak against substantial astrocytic lactate production and the ANLS hypothesis.

Although the present data do not hint at the oxidative degradation of glutamate in astrocytes, they fit in with and support this possibility. Transmembrane glutamate transporters, glutamate dehydrogenase (as the key enzyme channeling glutamate to the TCA), and mitochondria are spatially organized in a multiprotein complex, which is thought to facilitate direct glutamate access to the mitochondrium (Genda et al. 2011; Bauer et al. 2012). mGlu5 stimulation as proposed here would “announce” glutamate arrival and secure increased mitochondrial PDH activity when glutamate supply is increased; a feed‐forward regulation without a known mechanism until now. PDH increase is also key to complete glutamate oxidation. To be completely oxidized after the first TCA pass, the glutamate carbon chain needs to be transformed to pyruvate, before subsequent re‐introduction into the TCA cycle. PDH thus channels metabolites of both, glucose and glutamate to the TCA cycle, and the amount of glutamate undergoing oxidative metabolism increases depending on the extracellular glutamate concentration (between 100 and 500 μM) (McKenna et al. 1996). The attractive concept of the glutamate‐glutamine shuttle, where transmitter glutamate is quantitatively taken up by the astrocyte, converted to glutamine via glutamine synthetase and returned to the presynapse had to be modified, since both, glutamate transport (via Na^+^‐K^+^‐ATPase) and glutamine synthetase are ATP‐consuming. Once taken up into the astrocyte, glutamate is partly oxidized to generate the ATP required to shuttle back the other part of glutamate, that is, glutamate “pays its own way in astrocytes” (McKenna 2013). At the tripartite synapse, glutamate thus acts as a neurotransmitter, energy substrate, as well as signaling molecule in metabolic regulation, where mGlu5 stimulation triggers a mechanism that permits the complete glutamate oxidation (see above).

Further, the ATP drop induced by glutamate might appear small (12%) and thus easy to regenerate by the astrocyte. First, however, the ATP drop induced by extracellular glutamate might be attenuated because glutamate itself is oxidatively metabolized by astrocytes (see above). Second, our study aims at elucidating glial metabolic adaptation to the short‐term challenge of synaptically released glutamate only. During neuronal activity, several factors in addition to glutamate place a metabolic load on the astrocyte, and the actual contribution of extracellular glutamate to the overall load has remained an open issue. Thus, cultured astrocytes are energetically challenged in response to physiological levels of extracellular K^+^ and NH_4_^+^ (Lerchundi et al. 2015; Barros 2022), and their glycolytic rate is rapidly stimulated to > 300% by K^+^ (Barros 2022; Bittner et al. 2011; Lerchundi et al. 2015). By reductionist approach, extracellular K^+^ and NH_4_^+^ were excluded in our experiments (neuron‐free primary astrocytes; TTX in slice culture). When calibrating ATeam1.03YEMK fluorescence, we have previously estimated a baseline ATP concentration in astrocytes in organotypic slice cultures of 2.1 mM (Pape and Rose 2023; Lerchundi et al. 2020), suggesting that a decrease in ATP levels by 0.25 mM would correspond to a 12% decrease. Thus, this estimated ATP drop induced by extracellular glutamate reflects only a fraction of the astrocytic energy demand during synaptic activity. In addition, the presence of glucose in our cell culture medium (5.5 mM) has been shown to reduce the initial drop in [ATP] (Winkler et al. 2017).

With regard to a possible role in synaptic physiology, our study was designed to investigate and propose the mGlu5/PKCδ/PDH axis as a proof of principle. The actual processes in tissue occur in the time frame of several milliseconds to many seconds (in sustained neuronal activity) and in the extremely small volume of PAPs (Bergles et al. 1999). Considering the synaptic cleft and surrounding PAP appositions for simulations, it has been suggested that glutamate concentration in the synaptic cleft peaks at 1–2 mM, and that 100 or 500 μM prevail for several milliseconds (Matsui et al. 2005). We have applied glutamate concentrations of 100 μM (cell culture) and 500 μM (organotypic tissue slice culture). The actual contribution of the mGlu5/PKCδ/PDH axis proposed might be of relevance particularly under strong and sustained neuronal activity, since such condition entails both sustained extracellular glutamate levels and a high demand for ATP supply.

## Author Contributions

**Kiavasch M. N. Farid:** investigation, conceptualization, formal analysis, methodology. **Rodrigo Lerchundi:** investigation, formal analysis. **Christine R. Rose:** formal analysis, writing – review and editing, funding acquisition. **Amin Derouiche:** writing – original draft, investigation, conceptualization, methodology, writing – review and editing, formal analysis.

## Conflicts of Interest

The authors declare no conflicts of interest.

## Peer Review

The peer review history for this article is available at https://www.webofscience.com/api/gateway/wos/peer‐review/10.1111/jnc.70163.

## Data Availability

The data that support the findings of this study are available from the corresponding author upon reasonable request.
